# A Case of Malignant Biliary Obstruction with Severe Obesity Successfully Treated by Endoscopic Ultrasonography-Guided Biliary Drainage

**DOI:** 10.1155/2016/5249013

**Published:** 2016-09-06

**Authors:** Takashi Obana, Shuuji Yamasaki

**Affiliations:** Department of Gastroenterology, Kyojinkai Komatsu Hospital, Neyagawa, Japan

## Abstract

Here, we present a case of malignant biliary tract obstruction with severe obesity, which was successfully treated by endoscopic ultrasonography-guided biliary drainage (EUS-BD). A female patient in her sixties who had been undergoing chemotherapy for unresectable pancreatic head cancer was admitted to our institution for obstructive jaundice. She had diabetes mellitus, and her body mass index was 35.1 kg/m^2^. Initially, endoscopic retrograde cholangiopancreatography (ERCP) was performed, but bile duct cannulation was unsuccessful. Percutaneous transhepatic biliary drainage (PTBD) from the left hepatic biliary tree also failed. Although a second PTBD attempt from the right hepatic lobe was accomplished, biliary tract bleeding followed, and the catheter was dislodged. Consequently, EUS-BD (choledochoduodenostomy), followed by direct metallic stent placement, was performed as a third drainage method. Her postprocedural course was uneventful. Following discharge, she spent the rest of her life at home without recurrent jaundice or readmission. In cases of severe obesity, we consider EUS-BD, rather than PTBD, as the second drainage method of choice for distal malignant biliary obstruction when ERCP fails.

## 1. Introduction

Malignant biliary obstruction due to pancreatic head cancer is commonly encountered in clinical practice. Transpapillary biliary drainage by endoscopic retrograde cholangiopancreatography (ERCP) is usually attempted as the first decompression method. However, biliary cannulation sometimes fails during this procedure.As an alternative, percutaneous transhepatic biliary drainage (PTBD) or, more recently, endoscopic ultrasonography-guided biliary drainage (EUS-BD) has been performed. To date, the criteria for the optimal selection of these two salvage modalities have not been established. Here, we report a case of malignant biliary obstruction in a patient with severe obesity who was successfully treated by EUS-BD.

## 2. Case Presentation

**Figure 1:**
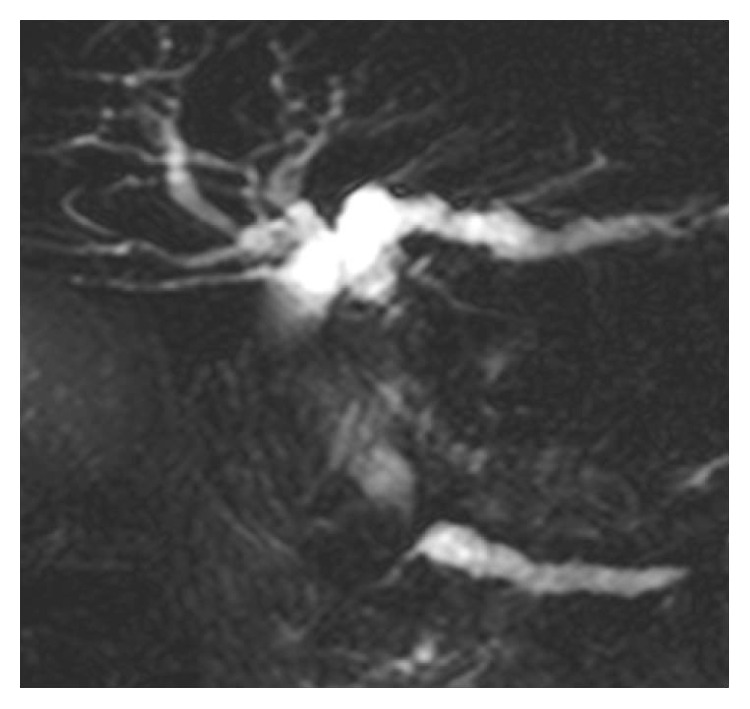
MRCP showed biliary obstruction in the lower portion and dilatation of the upstream bile duct.

**Figure 2:**
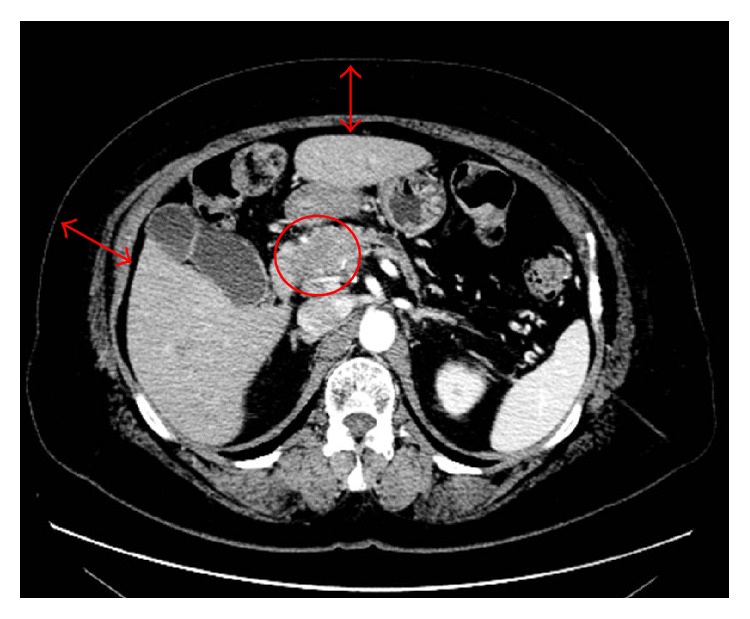
A hypoenhanced, irregular-shaped mass in the pancreatic head (*red circle*), as visualized by CT. The distance from the skin to the liver surface exceeded 4 cm (*red arrows*).

**Figure 3:**
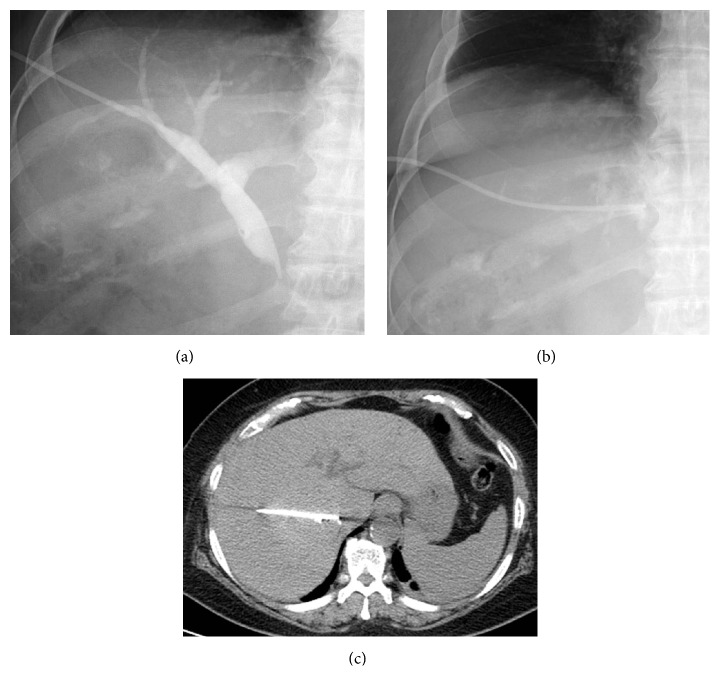
PTBD procedures. (a) Repeated PTBD by a right-sided intercostal approach was successful. (b), (c) After 2 days, the PTBD catheter was dislodged to the caudate lobe. Replacement of the catheter was unsuccessful.

**Figure 4:**
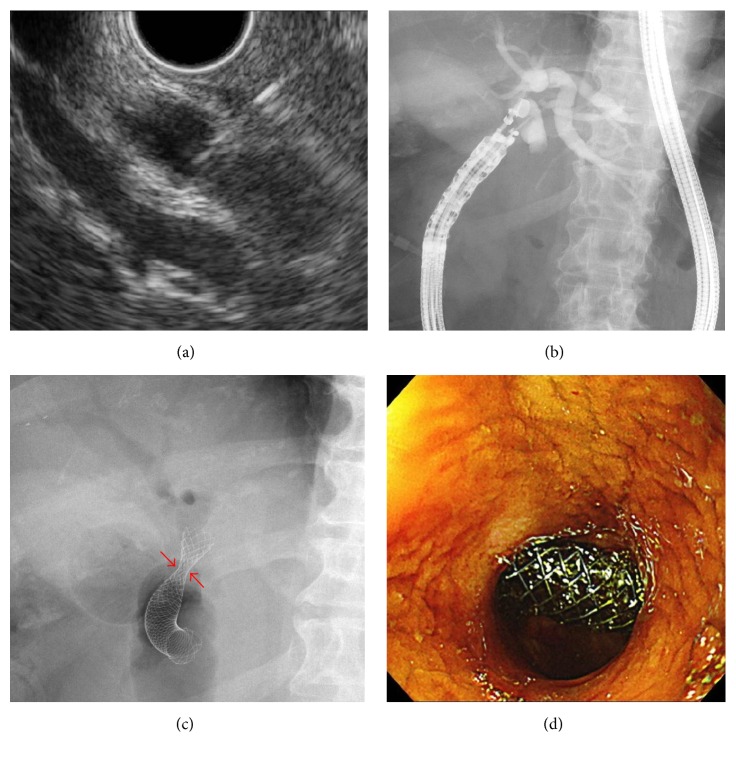
EUS-BD procedures. (a) The extrahepatic bile duct was visualized from the duodenal bulb using a curved linear array echoendoscope and punctured using a 19-G fine-aspiration needle. (b) The biliary tract was opacified with contrast medium. (c) A fully covered metallic stent was deployed through the choledochoduodenal fistula.* Red arrows* show the waist of the stent induced by the choledochal and duodenal walls. (d) Endoscopic view of the deployed stent observed from the pylorus.

A female patient in her sixties who had been undergoing gemcitabine chemotherapy for unresectable pancreatic head cancer was admitted to our institution for obstructive jaundice. She was severely obese with a body mass index (BMI) of 35.1 kg/m^2^ and had been diagnosed with diabetes mellitus. Laboratory test showed elevated total bilirubin level [5.5 mg/dL (normal range: 0.2–1.2 mg/dL)]. Magnetic resonance cholangiopancreatography (MRCP) revealed biliary obstruction in the lower portion and the dilatation of the upstream bile duct (Figure 1). On computed tomography (CT), a hypoenhanced, irregular-shaped mass was visualized in the pancreatic head. The distance from the skin to the liver surface was greater than 4 cm (Figure 2). After admission, therapeutic ERCP was attempted initially, but selective biliary cannulation was unsuccessful even with the pancreatic duct guidewire placement technique. On the second attempt, PTBD was performed by an experienced radiologist using the left-sided subxiphoid approach. Although the intrahepatic bile duct was visualized, catheter placement failed. A second PTBD procedure using the right-sided intercostal approach was successful. However, the patient developed biliary tract bleeding within 2 days, and the catheter became dislodged (Figure 3). Meanwhile, the patient's total bilirubin level had progressively deteriorated to 14.1 mg/dL. Therefore, EUS-BD was performed as a third drainage method after detailed informed consent. The extrahepatic bile duct was visualized from the duodenal bulb using a curved linear array echoendoscope (GF-UCT260; Olympus Medical Systems Co., Tokyo, Japan) and then punctured using a 19-G fine-aspiration needle (Expect 19 G; Boston Scientific, Natick, Massachusetts, USA). After visualizing the biliary tract by contrast medium injection, the puncture route was dilated with a bougie dilation catheter (ES dilator; Zeon Medical, Tokyo, Japan) and a balloon dilator (Max Force; 4 mm in diameter, Boston Scientific) over a guidewire (0.035-inch Jagwire; Boston Scientific). Finally, a fully covered metallic stent (WallFlex; 10 mm diameter, 6 cm length, Boston Scientific) was deployed through the choledochoduodenal fistula under fluoroscopy (Figure 4). The patient's postprocedural course was uneventful, and total bilirubin level gradually improved to 2.3 mg/dL. After discharge, she spent the rest of her days at home for 2 months without recurrent jaundice or readmission.

## 3. Discussion

Unsuccessful biliary cannulation during ERCP can occur in up to 5% of cases because of various factors. Several techniques including the use of papillotomes, placement of a pancreatic guidewire, and precut papillotomy have been used to improve cannulation rate[1–3]. PTBD is frequently performed as an alternative drainage method. In 2001, Giovannini et al. were the first to report the new technique of ultrasound-guided bilioduodenal anastomosis [4]. Since then, EUS-BD has been gradually gaining popularity [5–9] and it possesses several advantages over ERCP. Because EUS-BD is not performed transpapillarily, postprocedural pancreatitis is rare. Furthermore, the procedure can be used in cases with inaccessible papilla because of malignant duodenal stenosis or with complete lower bile duct obstruction.According to a systemic review [10], the cumulative technical success rate, functional success rate, and the adverse event rate associated with EUS-BD are 94.71%, 91.66%, and 23.32%, respectively. The adverse events commonly associated with EUS-BD are bleeding (4.03%), bile leakage (4.03%), pneumoperitoneum (3.02%), stent migration (2.68%), cholangitis (2.43%), abdominal pain (1.51%), and peritonitis (1.26%). Other studies have also reported that clinical success rates and complication rates do not differ significantly between EUS-BD and transpapillary stenting when the procedures are performed by expert endoscopists [11, 12]. However, because of the limited availability of echoendoscopes, this procedure cannot be introduced as the initial treatment in many institutions. Moreover, it is technically challenging, and the success rate may not be so high during the learning period [13]. Consequently, EUS-BD is basically regarded as an alternative to PTBD when ERCP fails and is largely selected at the discretion of the endoscopist involved. The optimal indications of EUS-BD have not yet been established, and customized devices for this procedure remain limited.

 Artifon et al. [7] compared EUS-BD and PTBD in different patient cohorts and reported that both procedures were technically and clinically successful in their prospective randomized study in which complication rates were 15.3% and 25%, respectively, without significant difference. Further, Khashab et al. [14] reported that although the technical success rate was higher in the PTBD group (100%) than in the EUS-BD group (86.4%), clinical success was equivalent (92.2% and 86.4%, resp.). However, adverse event rate was higher in the PTBD group (39.2%) than in the EUS-BD group (18.2%). Collectively, these results could not identify which of these two modalities was superior.

Complications associated with PTBD include catheter dislodgement, hemobilia, bile peritonitis, and pneumothorax. In the present case, catheter dislodgement resulted in repeated therapeutic procedures. From a retrospective point of view, EUS-BD should have been chosen as the second drainage method. PTBD catheter dislodgement is reported to develop in 4%–32% of cases [15–18]. Movement of the catheter during respiratory movement is considered as a reason for its dislodgement [17]. In this respect, the distance between the body surface and the intrahepatic bile duct appears as a risk factor for catheter dislodgement, because as the distance increases, PTBD catheter fluctuates more outside the liver. When a patient is severely obese, as was our patient in this case, the puncture route needs to be relatively long and thus potentially harbors a higher risk. On the other hand, EUS-BD is not influenced by the thickness of subcutaneous fat. Therefore, we consider that EUS-BD rather than PTBD is the preferable second drainage modality for such patients. The relationship between the length of a drainage route (or BMI) and the rate of catheter dislodgement needs to be examined further in future studies.

In conclusion, we consider that EUS-BD is a better second drainage method for distal malignant obstruction than PTBD in severely obese patients when ERCP fails. Optimal indications for the use of EUS-BD need to be further established by investigating more cases.
